# A Study on the Design of Embedded Visual Image Teaching Aids to Assist Young Children’s Cognitive and Fine Motor Development

**DOI:** 10.3390/jintelligence12100102

**Published:** 2024-10-14

**Authors:** Hua-Chen Lo, Tzu-Hua Wang

**Affiliations:** Department of Education and Learning Technology, National Tsing Hua University, Hsinchu 300193, Taiwan; huachenlo@hotmail.com

**Keywords:** visual graphic aids, cognitive development, fine motor skills

## Abstract

Visual development in infancy is crucial for establishing neural connections and enhancing the growth of the visual center. Adequate visual stimulation supports cognitive learning, helping children integrate images, colors, and shapes. This research examines the design and use of a visual image teaching aid to guide children in exploring image cognition and developing fine motor skills. This study involved 70 kindergarten teachers who participated in a questionnaire survey about 60 children aged from 5 to 6 years old. The results show that visual graphic aids effectively promoted coordination, control, and the integration of visual and fine motor skills in children. Furthermore, these aids supported the development of spatial and environmental relationships through hands-on activities.

## 1. Introduction

The development of visual perception in preschool children is a crucial aspect of their learning process. Warren (1993) proposed a hierarchical model for the development of visual perception skills, emphasizing the integration of visual reception and cognition to form functional vision. Functional vision comprises various elements, including visual acuity, attention, search, graphics, memory, and cognition, all of which contribute significantly to a child’s overall sensory perception. In young children, vision accounts for from 75% to 85% of sensory input, followed by hearing and touch, making it the most dominant sense in early learning.

Visual–motor integration (VMI), which refers to the unification of visual and motor coordination, is fundamental for children’s exploration of their environment. VMI not only facilitates the reception and processing of visual information, but also plays a key role in the development of sensorimotor skills and cognitive abilities. As children engage in activities that involve both visual perception and motor skills, they develop an essential foundation for learning and interacting with their surroundings.

Kephart (1971) Young children’s visual exploration is inherently linked to a variety of cognitive processes. Kephart’s perceptual-motor theory emphasizes that visual perception and perceptual-motor training are vital for enhancing cognitive learning in children. For example, when children observe the shapes and characteristics of objects, they connect these visual cues to their everyday experiences, developing an understanding of spatial relationships. These skills are closely associated with body coordination and control, as seen when children arrange building blocks based on patterns, background relationships, ad image search. Therefore, children experiencing visual developmental challenges may struggle with visual–motor integration, leading to difficulties in object recognition, detailed perception, and hand-eye coordination as these skills are all closely related to body movement coordination and control.

Research has shown that visual perception in preschoolers significantly influences higher-level cognitive functions and can predict future literacy development (O’Connor and Jenkins 1999). Recognizing this, this study draws on Gibson’s ecological optics theory, which highlights the importance of cognitive learning, learning preparation, and intrinsic motivation in learning activities (Gibson 2014). By providing preschool children with rich perceptual experiences, visual image teaching aids can enhance their learning experience, support cognitive development, and lay a solid foundation for comprehensive growth. Additionally, it is important to consider how children’s learning experiences may be influenced by factors such as socialization, gender differences, and adult attitudes. Research indicates that these factors can shape children’s game behaviors and preferences (Ruble et al. 2006). Therefore, this study also aims to explore whether gender differences impact how children interact with visual images and spatial templates.

The objectives of this study are threefold: first, to design a visual imagery teaching aid that enhances children’s conceptual knowledge and cognitive skills; second, to compare the perceived effectiveness of the developed aids with traditional visual imagery teaching aids, as reported by educators and childcare professionals; and third, to assess children’s cognitive development and fine motor learning before and after using these teaching aids.

To address these objectives, this study seeks to answer the following research questions:

1. What are the perceived differences in effectiveness and functionality between traditional visual aids and the aids developed in this study, as reported by education and healthcare professionals?
2. How does the use of the developed teaching aids influence the cognitive development and fine motor learning of children, based on assessments conducted before and after their use?

## 2. Literature Review

### 2.1. The Concept and Theoretical Foundation of Visual Cognition in Young Children

**Figure 1:**
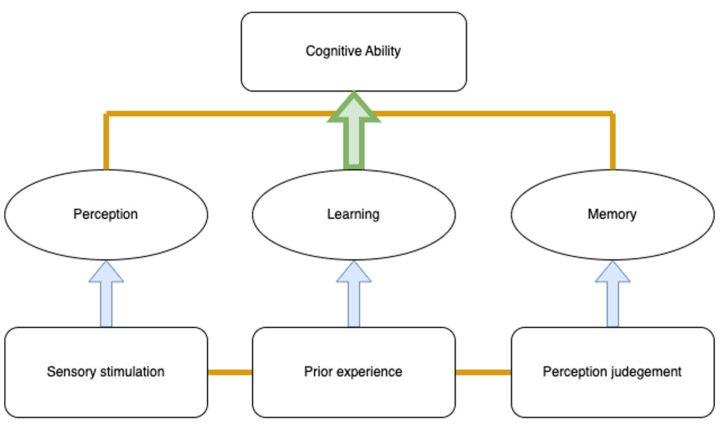
Relationship between sensory perception and learning.

The development of visual and motor skills plays a significant role in a child’s learning process. In this study, visual teaching aids were designed to facilitate children’s interaction with visual information, allowing researchers to observe their learning performance. Since vision is one of the main ways children use to explore and gather information from their environment, this visual input may help them build a stronger foundation of conceptual cognition, thus enabling them to understand the meaning conveyed by images and connect these images with specific concepts. For instance, exposure to different colors and shapes encourages children to observe, identify, manipulate, and respond to various characteristics within their environment. Through this process, children gradually develop the basic structure of conceptual cognition. Figure 1 below illustrates the relationship between sensory perception and learning, demonstrating how children construct cognitive abilities through visual exploration and the interaction between perception and learning.

According to the theory of cognitive representation systems, an individual’s cognitive representation of external stimuli is a continuous psychological process (Bruner et al. 1966; Levitin 2002; Heft 2020; Hommel 2019). Within the learning context, the structured, systematic, and sequential organization of cognitive processes and teaching materials can effectively stimulate young children’s motivation for active learning. Bruner’s framework outlines three phases of cognitive development in young children: motor representation, pictorial representation, and symbolic representation (Makransky and Petersen 2021; Takaya 2015).

During the preschool stage, children primarily acquire information through sensory perception. Their awareness of visual images progresses from concrete to abstract, gradually transitioning into cognitive learning through the use of graphics and symbols. Since young children have not yet developed the skills of reading and writing, they often represent objects and concepts in their lives through drawings and images. Therefore, it is essential to provide progressive manipulative learning materials that enable children to engage in hands-on activities and transform their learning experiences into cognitive learning processes. This approach facilitates their understanding and conceptualization.

Table 1 below provides an overview of Bruner’s theory of cognitive representation systems, which outlines the stages through which children process information and develop their cognitive abilities.

The Complete School of Psychology posits that the comprehensive and structured nature of visual images, encompassing shapes and forms, can facilitate learners in establishing direct experiential connections with phenomena (Feest 2021). By organizing visually collected image information and engaging in hands-on manipulative experiences, learners can explore the interconnections and relationships between objects, thereby constructing their cognitive understanding. For instance, through analyzing image compositions, decompositions, object order, and rules, children can identify, classify, and categorize relationships between wholes and parts, as well as analyze cause and effect. These processes foster the development of systematic logical thinking, knowledge construction, higher-level thinking, mental feedback, and problem-solving abilities.

Furthermore, the Gestalt school of psychology emphasizes that the human brain operates based on holism. In the context of visual perception, this means that an image is perceived as a whole rather than merely a sum of its parts. Our perception of an object is not only shaped by its external features such as shape, color, and size, but is also influenced by our past experiences and impressions. This perceptual experience is closely linked to brain activity and plays a significant role in how young children form concepts.

The Gestalt psychology framework is based on four foundational principles: Emergence, Reification, Multi-stability, and Invariance. These principles are key to understanding human visual perception and were used to guide the visual design aspects of this study:

1. Emergence: Our eyes seek to identify an object’s outline, and by comparing it with past memories, we can quickly recognize the object. This principle represents the holistic nature of human vision.
2. Reification: When our vision is stimulated, and we interpret spatial information from the external environment, our brains tend to “fill in” missing details, creating a complete message for understanding observed objects.
3. Multi-stability (Organization): If an object has more than one possible interpretation, our brains can only process one interpretation at a time, although we can switch between different interpretations.
4. Invariance: This principle highlights the constancy of our vision. Regardless of how an object changes (e.g., deforms, rotates, enlarges, or shrinks), we can still recognize it through its outline or features.

To summarize, the Gestalt psychology believes that learning is a process of visual and cognitive reorganization where knowledge and skills are developed through problem-solving during experiential learning. Engaging with Gestalt patterns not only fosters creative thinking, but also enhances problem-solving abilities.

**Figure 2:**
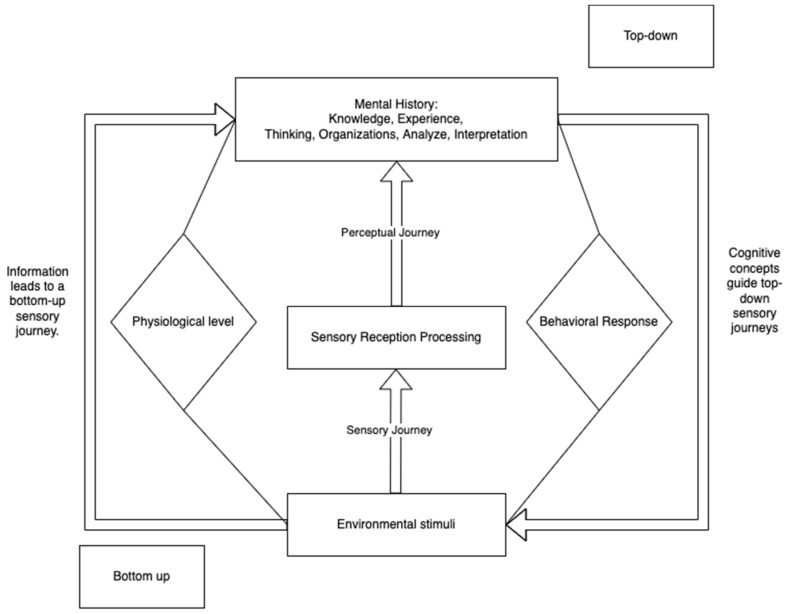
Zimbardo and Gerring (2009), cognitive process.

The visual perception process is a systematic approach to processing environmental stimuli through sensory perception (Levitin 2002; Zimbardo and Gerring 2009). This process involves the coding and structuring of information into short- and long-term memory. In comparison, the top-down conceptual learning process is influenced by the individual’s motivation, experiences, and knowledge, interacting with the environmental information. As shown in Figure 2.

Lastly, Robert J. Sternberg’s triarchic theory of intelligence offers additional insight into human cognitive abilities by identifying three dimensions: combinational intelligence, experiential intelligence, and adaptive intelligence (Sternberg 1984). Research indicates that an individual’s experiences significantly impact the development of cognitive skills, particularly in thinking, planning, and execution. This cognitive development occurs through mental representations, cognitive processes, and the integration of declarative and procedural knowledge, combined with an awareness of environmental interactions.

By synthesizing the aforementioned theoretical foundations that emphasize cognition, knowledge, and experiences within the cognitive learning thinking process, children can foster a connection with their internal mental abilities. Through a progression from simple to complex, concrete to abstract, and action to symbol, children engage in the process of exploring thinking and reasoning. This generates motivation and enhances their active learning, ultimately leading to positive learning outcomes.

### 2.2. Establishing the Foundation for Visual Learning in Early Childhood from a Dynamic Systems Perspective

Early childhood years play a crucial role in motor development, as they involve the integration of various developmental and learning abilities through both gross and fine motor skills. According to Gallahue, motor skills can be categorized into locomotion skills, manipulation skills, and stability skills (Cleland and Gallahue 1993). These fundamental movement skills serve as the building blocks for young children’s overall movement development (Liu 2018). Through the practice of dynamic (gross motor) and static (fine motor) movements, children can enhance their physical movement capabilities and refine their control over their bodies (Trevlas et al. 2003). Gallahue and Ozmun emphasize that movement development is influenced by the interaction between the individual, the specific movement being performed, and the surrounding environment (Gallahue and Ozmun 2006). This dynamic systems perspective emphasizes how these factors interact to influence children’s motor development.

From the perspective of Dynamic Systems Theory (DST), movement development is shaped by environmental stimuli and the purpose behind actions (Thelen and Smith 1995). The motor and nervous systems work together, creating perceptual experiences through internal control and self-initiated movements. Signals are sent from the central nervous system to the skeletal muscles to perform movement tasks, while proprioception provides feedback to the brain about body position and movement. Different movement patterns introduce varying levels of flexibility within the neural system. As these patterns are gradually refined, coordination and control improve, leading to the development of new motor skills.

Spatial awareness, which plays a key role in motor development, is closely related to how visual representations are formed. A child’s motor development is influenced by both perceptual stimuli from their environment and their ability to integrate cognitive information (Siegel and White 1975). Therefore, children will frequently explore spatial directions based on their own perspective and rely on visual cues to recognize and navigate their surroundings (Leikin and Sriraman 2022).

In this study, perceptual awareness theory serves as the foundational basis for constructing a learning framework that promotes visual–motor integration through the use of visual graphic aids. As proposed by Baddeley and Hitch, visual space encompasses both visual and spatial information, which is crucial for processing attributes such as color, shape, and spatial relationships within the external environment (Baddeley and Hitch 1974). Spatial visualization involves the mental transformation and manipulation of visual elements, where processes such as the deconstruction, organization, synthesis, and overlapping of images contribute to the development of spatial cognition (Montello et al. 2004). To facilitate cognitive development in children, physical manipulative interfaces offer intuitive cues that guide them in recognizing and interpreting the relationships and intentions conveyed by visual imagery. This hands-on interaction creates an optimal environment for cultivating fine motor skills, thereby enhancing coordination and control as part of the visual–motor integration process. Visual images, when combined with these interfaces, connect dynamic spatial perceptual information, thereby stimulating children’s intrinsic motivation to engage in exploratory learning.

As children interact with these visual aids, they undergo cognitive processes including perceptual awareness, analytical reasoning, recognition, and the evaluation of appropriate action responses. This engagement fosters key learning outcomes, such as self-regulation, coordination, heightened awareness, and reflective thinking. By integrating visual–motor skills within a structured and supportive learning environment, children systematically refine their fine motor coordination and control. Additionally, as they participate in activities that require the organization and synthesis of information, their problem-solving capabilities are further developed, thereby enhancing their preparedness for subsequent learning experiences.

In summary, the visual imagery teaching aids designed in this study provide children with direct perceptual experiences and leverage environmental affordances to support sensory information processing in the brain. This approach enables children to develop mental models that correspond to elements in their environment, fostering their intrinsic motivation to explore and act. The interface design of the teaching aids incorporates both real and perceived affordances, encouraging children to consider the physical aspects of object manipulation as well as the meaning and logic behind images and symbols. This guided exploration promotes the development of perceptual and motor skills, refining their cognitive and motor abilities and establishing a solid foundation for proficient visual and motor skills. Ultimately, this approach enhances cognitive learning and motor skills, laying the groundwork for effective visual–motor integration.

## 3. Research Methodology

### 3.1. Design Concepts of Visual Graphic Teaching Aids

Visual perception and movement coordination skills are interconnected, as visual movements rely on the coordination between visual perception and physical actions (Sutton et al. 2011). Warren (2011) proposed the concept of the Hierarchy of visual perceptual skill development, dividing visual perception into two parts: visual reception and visual cognition. These components work together to form what is known as functional vision. Visual cognition can then be further divided into four key elements: scanning, visual attention, visual memory, and visual discrimination. In this study, an extensive review of traditional teaching toys and the relevant literature on the market revealed that most existing visual teaching aids for children are typically in the form of picture card-based tools. These aids, while useful, tend to have a limited scope and often need to be supplemented with additional teaching materials to provide comprehensive training and support.

Schneekloth observed that children with visual impairments demonstrate fewer gross motor and social play behaviors (Schneekloth 1989). This behavior is not primarily due to innate sensory deficits, but rather is due to a lack of experience in interacting with their environment. He emphasized that the absence of a suitable play environment restricts these children’s opportunities for exploration. Tröster and Brambrin (1970) found that delayed onset of symbolic play behavior in visually impaired children could not be attributed solely to developmental delays in representational intelligence, as discussed by (Verver et al. 2020). The issue may be that traditional symbolic toys, such as toy cars and dolls, often fail to provide realistic representations of real-world objects or people, lacking tactile and auditory elements. These toys, being mere miniaturized versions, are not always appropriate or engaging for children with visual impairments.

There are many forms of traditional teaching aids currently on the market. One of the most common teaching aids comes in the form of simple matching games or puzzles. These aids encourage children to focus on clues within the images to combine or compare different shapes, thereby enhancing their visual concentration. Additionally, these type of teaching aids often include exercises that support the development of logical reasoning, calculation skills, observation, identification, and the concept of numbers. Next, the researchers found another type of visual teaching aid that involves using self-created materials. For example, maze-like pictures can guide children in exploring visual information and train their visual tracking skills. Furthermore, there are also teaching aids intended for home-based training, such as reward cards that recognize the completion of homework or specific activities. These aids not only help children practice communication through visual prompts and simple language, but they also reinforce memory and encourage positive behaviors. While there are many commercially available teaching aids on the market, the types, functions, and methods of operation tend to be relatively basic. The visual diagrams provided are usually concrete and intuitive, but lack guidance in developing a conceptual understanding of more abstract image symbols. Additionally, they do not provide opportunities for peer collaboration and joint discussions which enable problem solving, which are crucial for fostering cooperative learning and deeper cognitive engagement. Through the process, we can establish specific concepts of objects and effectively produce movement coordination to smoothly carry out games and activities, which helps to improve children’s cognitive learning ability (Baumgartner et al. 2015; Huang et al. 2020).

As part of this study, researchers identified and reviewed standardized tests that are commonly used to assess the development of children’s visual–motor integration skills. It was observed that most of these assessments require individualized administration to accurately evaluate each child’s visual movement abilities. The assessment methods are designed to measure various aspects of visual integration skills. Table 2 is a list of standardized tests frequently used to assess visual integration.

Based on the standardized test above, the researchers of this study identified that traditional game materials and operating methods often vary and do not always provide an environment that meets the needs of young children. In response, the teaching aids developed in this research have been designed to accommodate the diverse aspect of children’s game development and interactive behaviors. These aids integrate visual graphics and card activities with motor skill development to offer a more comprehensive and effective learning experience.

This study applies the principles of Gestalt Theory, first introduced by Max Wertheimer and colleagues in 1912 (Wagemans et al. 2021) to create visual images that reflect the concept of dynamic wholes and the principles of ideal form. By drawing on cognitive psychology, the study emphasizes the importance of meaningful perception in the visual construction of cognitive processes. Consequently, the visual images used in the teaching aid are designed to convey cognitive experiences related to shapes, forms, dynamics, and holistic exploration. The visual image teaching aids developed and used in this study consist of a main structural module and visual image design components. These teaching aids have been recognized for their innovation, having received two patents and having been awarded the gold medal at both the 2019 Tokyo Invention Competition and the 2023 IIIC International Invention Competition. Additionally, the teaching aid has been granted an invention patent in the Republic of China, with patent number I817589.

The primary structure of the teaching aids developed in this study integrates multiple visual and motor operating methods. It utilizes an embedded picture approach for visual image conversion, allowing children to freely explore shapes and forms through actions such as layering, stacking, displacement, pulling, and rotation. The teaching aid aims to foster cognitive learning by enabling children to engage with concepts and experiences in a hands-on manner. Children can follow the patterns and colors displayed on the instructional images to explore the dynamic transformation of visuals. Additionally, through the use of situational cards and relational clues, they learn to identify subjects and specific graphic details. As they progress through this visual cognition process, children gradually develop an understanding of concepts such as repetition, sequencing, graphic transformation, and spatial positioning.

**Figure 3:**
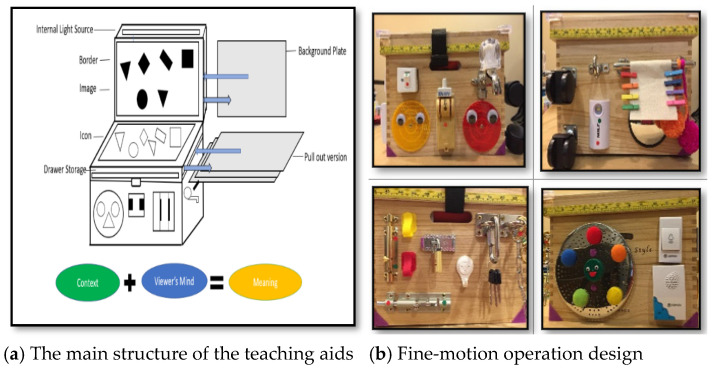
Embedded visual teaching aids include main structure and fine motor operation design.

**Figure 4:**
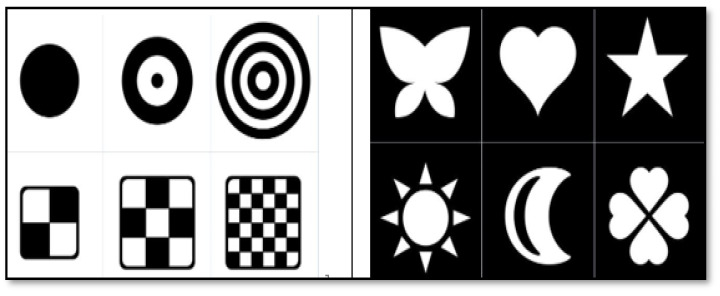
Traditional visual image teaching aid picture cards.

The design of the embedded visual aid used in this study is illustrated in Figure 3 below. Figure 4 shows a traditional visual picture card, which usually provides visual recognition exercises for young children. It has a relatively simple function and does not provide opportunities for operational practice.

### 3.2. Design Framework for Visual Graphic Teaching Aids

In this study, the design of visual image teaching aids is grounded in the framework of the following six elements of visual perception: Form Constancy, Visual Closure, Figure–Ground, Position in Space, Depth Perception, and Topographic Orientation. Additionally, the eight principles of Gestalt Theory serve as the scaffolding for the visual image content of the teaching aid. These principles encompass the principles of similarity of shapes, proximity, continuity, closure, the relationship between shapes and backgrounds, common orientation, symmetry, and common direction (Chen and Ma 2022). By utilizing embedded visual images, children are exposed to visual images with diverse functions and characteristics, facilitating their engagement and utilization of visual senses for perceiving spatial information from the external environment. This approach helps children recognize the form of individual images and explore the conceptual connections among graphic elements based on their previous experiences. Furthermore, it enables children to read and interpret image elements and their relationships. For instance, during manipulative tasks, children are guided to identify similar elements and connect their understanding of image features. When observing and categorizing graphic symbols based on their shapes, children can discern commonalities and regular patterns across different colors and shapes. Through hands-on learning experiences, children’s sensory exploration is actively stimulated, deepening their comprehension of the organization and structure of pictorial symbols, shapes, and colors. This, in turn, encourages children to contemplate the interconnectedness of cognition and internal resonance, bridging the external stimuli with their internal thought processes.

The teaching aids in this study include:

1. Visual attention game board: This board uses patterns on visual cards to stimulate the interaction between vision, brain cognition, and spatial perception, guiding children to develop an understanding of object concepts.
2. Visual memory game board: Children practice matching images through memory-based operations. This activity helps them confirm the location of objects based on the icons displayed on the cards, reinforcing their memory skills.
3. Visual discrimination game board: Through the manipulation of picture cards, children learn to identify spatial relationships, distinguish differences in themes and object details, and engage in activities such as identification, pairing, and classification. These exercises help connect visual stimuli with memory, supporting cognitive development in young children.
4. Visual imagination construction games: Using finger puppets and situational picture cards, children practice storytelling and develop their oral expression skills, fostering creativity and imagination.
5. Visual pursuit game: This game encourages children to use visual tracking by working with graphics and symbols in various colors. They can classify, identify, or trace paths on the game board, enhancing their visual processing abilities.
6. Fine motor operation practice: Children engage in exercises that involve grasping, holding, twisting, turning, pulling, pressing, and other fine motor skills. These activities are integrated with visual–motor development exercises, allowing children to practice coordinated actions using the teaching aids.

Table 3 provides a detailed description of the design framework for visual image teaching aids, as well as the strategies employed for graphic design and teaching in this study.

### 3.3. Research Design

This study utilized a combination of questionnaires and structured observations to collect and analyze data. The “Questionnaire on the Functions and Characteristics of Visual Imaging Teaching Aids” was employed in the questionnaire survey to investigate the differences in perceptions regarding the functions and characteristics of traditional teaching aids versus the teaching aids developed in this study. The implementation method involved participants completing the questionnaire after using both the traditional teaching aids and the visual image teaching aids developed in this study. The responses from the questionnaires were then compared and analyzed.

For the structured observations and assessments, teachers observed the learning progress of the children before and after utilizing the visual imagery teaching aids. Structural assessments were conducted to evaluate the children’s performance, and the results of both assessments were analyzed to gain insights into the effectiveness and progress made by the children throughout the learning process.

### 3.4. Participants

A total of 70 in-service teachers were invited to participate in the questionnaire survey. Additionally, four experienced kindergarten teachers were responsible for conducting the structured assessments, observing the learning progress of 60 children aged 5–6 years old both before and after the teaching sessions. These participants were selected to ensure a diverse and representative sample for the study.

### 3.5. Research Instruments

This study used questionnaires and structured observation and evaluation to collect and analyze data. In terms of questionnaire survey, the Visual Image Teaching Aids Functions and Characteristics Questionnaire was used to investigate the current teaching and nursing staff to understand the differences in their feelings about the functions and characteristics of traditional teaching aids and the teaching aids in this study. The specific implementation method is as follows: First, let Participants first use traditional teaching aids and then fill out the Questionnaire on Functions and Characteristics of Teaching Aids], and then use the visual image teaching aids in this study, and then fill out the [Questionnaire on Functions and Characteristics of Visual Image Teaching Aids, and then compare and analyze the responses. In terms of structural observation and evaluation, teachers observe children’s learning before and after the use of visual image teaching aids, conducting structural evaluations, respectively, and then analyze the results of the two evaluations before and after to understand the children’s performance before and after learning, and the effectiveness of each.

1. Questionnaire on the Functions and Characteristics of Teaching Aids

The researchers developed a questionnaire on the functions and characteristics of teaching aids based on the design of visual image teaching aids, as outlined in the literature. The questionnaire aimed to investigate users’ perceptions of the functions and characteristics of the teaching aids. The development process involved obtaining feedback and revisions from five experts regarding the content of the questionnaire. A pilot test was conducted with 100 education and childcare professionals. The questionnaire consists of two dimensions, functions and characteristics, comprising a total of 12 items. The estimated completion time is approximately 15 min. A five-point Likert scale was used for responses, with options ranging from “Very Important” to “Not at all Important,” with corresponding scores of 5 to 1. The pilot test participants consisted of 100 education and childcare professionals, with 43% aged 21–30, 26% aged 31–40, 28% aged 41–50, and 3% aged 51 or above. In terms of occupation, 43% were kindergarten teachers, 54% were daycare center staff, and 3% were parent–child center staff.

Pilot test data analysis results: (1) Homogeneity test: The correlations between the items and total scores ranged from 0.77 to 0.59, all exceeding 0.35, indicating the retention of all items. (2) Critical ratio (CR): The CR values were above 3.5, indicating the discriminative power of the items. The results show that the CR values of all items ranged from 6.97 to 12.88, indicating that all items were retained. (3) Factor analysis: Exploratory factor analysis yielded two factors. The Kaiser–Meyer–Olkin (KMO) value of the questionnaire was 0.86, indicating good sampling adequacy. The analysis identified two factors: the first factor had an eigenvalue of 6.44, explaining 53.68% of the variance, and was named “Characteristics of Teaching Aids”. The second factor had an eigenvalue of 1.23, explaining 10.21% of the variance, and was named “Functions of Teaching Aids”. These two factors accounted for a cumulative variance of 63.89%. See Table 4 for a summary of the factor analysis. (4) Reliability analysis: Internal consistency was examined using Cronbach’s α coefficient for both the overall scale and subscales. The subscale “Characteristics of Teaching Aids” demonstrated a reliability of 0.86, the subscale “Functions of Teaching Aids” demonstrated a reliability of 0.88, and the overall scale demonstrated a reliability of 0.91. All reliabilities exceeded 0.80, indicating the good internal consistency of the questionnaire. Overall, the questionnaire demonstrated good validity and reliability in assessing users’ perceptions of the functions and characteristics of the visual image teaching aids.

2. Structured Observational Assessment

A structured observational assessment was employed to examine the learning outcomes of young children before and after using the visual image teaching aids in this study. Over a period of 12 weeks, four teachers guided the children in the use of the teaching aids. Two structured assessments were conducted, one before and one after the use of the visual image teaching aids, focusing on four dimensions: (1) graphic recognition, (2) graphic comprehension, (3) spatial thinking, and (4) fine motor skills. To ensure the validity and reliability of the assessment, the items were reviewed and modified by experts in the field of early childhood education to ensure their appropriateness. The opinions of the experts indicated a consensus with an agreement index of >0.8, demonstrating the content validity of the assessment.

The teachers observed the children’s actual performance during the learning activities and recorded their observations using a checklist-based assessment. The scoring system ranged from 1 (not yet developed) to 5 (highly proficient), reflecting the level of the children’s performance. To ensure fairness and objectivity in the assessment, different teachers conducted the pre- and post-assessments. After completing the assessments, the teachers discussed the observations with the classroom teacher to confirm the consistency between the children’s actual performance and the assessment. The data collected from the assessments were then compiled and analyzed using a univariate analysis of covariance (ANCOVA) to examine the statistical differences.

The structured observational assessment provided valuable insights into the children’s learning outcomes and their development in various dimensions related to the use of visual image teaching aids. The use of multiple observers and the collaboration with the classroom teacher enhanced the validity and reliability of the assessment process. The collected data will be further analyzed to examine the impact of the teaching aids on the children’s learning progress.

## 4. Results

### 4.1. Comparative Analysis of the Effectiveness of Traditional Teaching Aids and Visual Aids

To compare the effectiveness of traditional teaching aids and the visual imagery teaching aids developed in this study, the results of the questionnaire survey on the characteristics and functions of both types of teaching aids were analyzed. The analysis revealed that the visual imagery teaching aids in this study demonstrated higher mean scores for both features and functions compared to the traditional teaching aids. The descriptive statistics of the characteristics and functions of the toys are presented in Table 5 and Table 6, respectively.

The findings suggest that the visual imagery teaching aids designed in this study possess enhanced features and functions that have the potential to facilitate children’s learning and development. The comparison between the two types of teaching aids provides valuable insights into the advantages of utilizing visual imagery in educational settings. These results will contribute to a deeper understanding of the impact and effectiveness of visual aids in early childhood education.

This study further conducted a dependent sample t test on the total average of the subscales of “Features of Teaching Toys” and found that the *p*-value reached a significant level of 0.05, indicating that the visual image teaching aids in this study can satisfy children’s exploration and curiosity and help children learn. The ability to think and solve problems has a significantly better effect. It is also significantly better than traditional teaching toys in terms of connecting children’s life experience and guiding children to explore. Its effect size (effect size) Cohen’s d value is 0.82, which is a big effect size. The total mean of the subscale of “Function of Teaching Toys” was then subjected to a dependent sample t test, and the result found that the *p*-value reached a significant level of .05, indicating that the design of the teaching toys is creative, has diverse play methods, and improves children’s fine movements and self-care. In terms of ability development, providing children with different visual and tactile stimulation and visual cognitive learning functions is significantly better than traditional educational toys. Its effect size, Cohen’s d value, is 0.82, which is a big effect size.

### 4.2. Differences in Educational Care Providers’ Views on Features and Functions of Teaching

In this study, a one-way analysis of variance (ANOVA) was conducted on the “features of educational toys” among 70 childcare workers of different ages, educational levels, types of centers, years of experience, and ages of children, and the results showed that there were no significant differences in any of the items. However, only the F-value of 4.63 for different years of experience was found to be significant at 0.05 for “features of toys”. The mean score for “features of toys” was found to be significantly higher for those with less than five years of experience than those with more than five years of experience. Table 7 and Table 8 below illustrate the results of the Characteristics and Functions of Educational Toys subscales, respectively.

The results of Table 4, Table 5, Table 6, Table 7 and Table 8 show that the visual graphic aids designed in this study provide more diverse visual and graphic cognitive learning than traditional teaching toys in terms of features and functions and can arouse children’s interest in exploring and learning, enable children to practice thinking and problem solving during the manipulative process, and promote children’s fine motor development. There were no significant differences in the views of educational service providers on the characteristics of educational toys from different backgrounds. There were no significant differences in the “functions of educational toys”, except that those with less than five years of experience scored significantly higher than those with more than five years of experience, while there were no significant differences in the “functions of educational toys” among the other background variables. It shows that those with less than five years of work experience attach more importance to the function of teaching toys.

### 4.3. Structural Observation of Children’s Visual Graphics Learning Assessment Results

Before and after the teacher guided the children to manipulate the visual graphic aids for learning, structured observation was used to collect data on the four dimensions of pattern recognition, pattern comprehension, spatial thinking, and fine motor movements, and it was found that the children’s mean scores on the post-test of the four dimensions of the visual graphic aids were significantly better than those on the pre-test. Table 9 below shows the descriptive statistics of the pre-test and post-test of the visual graphic learning assessment. The results of the t tests of the dependent samples were all significantly different, and the post-test scores were all higher than the pre-test scores, which means that the children’s ability in pattern recognition, pattern understanding, spatial thinking, and fine motor moments improved after learning with the visual graphic aids of the study, and the effect sizes of the aids ranged from 0.40 to 0.49. According to Cohen’s (1988) suggestion of low (Cohen’s d = 0.2), medium (Cohen’s d = 0.5), and high (Cohen’s d = 0.8) effect sizes, the results of the present study were close to medium effect sizes.

In this study, we further compared the differences in learning effectiveness between male and female children by using a one-way covariate analysis with the pre-test scores as the covariate, gender as the fixed factor, and “pattern recognition”, “pattern comprehension”, “spatial thinking”, and fine-motor scores as the dependent variables. First, the intra-group regression coefficient homogeneity test was conducted. From Table 10, it can be seen that the intra-group regression coefficient homogeneity test of the four items of the visual image assessment showed that the F-value of “pattern recognition” was 1.13, with a *p*-value of .289; the F-value of “pattern understanding” was 0.43, with a *p*-value of .514; the F-value of “spatial thinking” was 0.21, with a *p*-value of .651; and the F-value of “fine motor movements” was 0.36, with a *p*-value of .549. None of the four items reached a significant level, which was consistent with the basic assumptions, so the analysis of covariates continued. The results of the covariate analysis are shown in Table 11. After removing the effect of the covariate (pre-test scores), there was no significant effect of gender on the post-test scores, which indicated that there was no significant difference in the effectiveness of the visual graphic aids developed in this study among children of different genders.

Table 11 shows the results of the covariate analysis. It shows that after removing the influence of covariates (pre-test scores), the F-value and *p*-value of gender on the performance of the four post-test assessments did not reach significant differences, indicating that gender does not affect the learning effect of visual image teaching aids. Regarding gender, some researchers found related studies and found that the ability to encode and distinguish pictures and text increases with age, but another related study showed that gender has no significant predictive effect on logical thinking ability (Cohen 1988; Ghatala and Levin 1973).

In summary, the above results showed that after operating the visual imagery aids designed in this study, the children’s performance in the four assessment items of pattern recognition, pattern comprehension, spatial thinking, and fine motor movements in the post-test was better than that in the pre-test, which indicated that the use of the visual imagery aids in this study was effective in learning, and that there was no significant difference in the effectiveness of the aids in assisted learning between different genders of the children. In other words, this study enables us to understand that the learning process of young children through the senses not only relies on the physiological maturity of the individual, but also promotes their internal motivation through interaction with the environment and gradually facilitates their cognitive structure and mental operations using visual images and spatial models. The results of this study also echo the interaction between the three stages of cognitive development according to Bruner’s theory of the representational system and provide structured learning through manipulative learning using the visual image teaching aids. Through the use of manipulatives, we can provide structured learning bridges to promote children’s intuitive thinking for exploration and discovery and utilize visual–analogical integration to unify the conceptual links and developmental balance in cognitive learning, which can further help children to lay good foundations for learning.

## 5. Discussion and Conclusions

### 5.1. Educational Service Personnel’s Views on Visual Graphic Aids and Traditional Teaching Aids in This Study

This study analyzed the results of the questionnaire on the characteristics and functions of traditional teaching aids and the visual image teaching aids in this study. The results found that the average features and functions of the visual image teaching aids in this study were higher than those of traditional teaching toys. The characteristics of the visual image teaching aids in this study can provide children with real-life experience and arouse children’s interest in learning. The overall design is from easy to difficult, and can guide children to explore learning opportunities and provide children with the ability to learn, think, and solve problems, and it is of educational significance, practical, reusable, and economical, and is better than traditional single teaching aids. Educational care staff also believe that the creative design of teaching toys can enhance the development of children’s fine motor skills.

The design also introduces more diverse and rich play methods, providing children with different visual and tactile stimulation operations and exercises to cultivate children’s development of their fine motor and self-care abilities.

### 5.2. Children’s Performance in Structural Assessment of Operating Visual Image Teaching Aids

Based on the teacher’s observations and evaluations of the children’s performance before and after using the teaching aids, it was found that their learning outcomes improved in four key areas: pattern recognition, pattern understanding, spatial thinking, and fine motor skills. The children’s average performance after the intervention was significantly better than before, indicating that the visual imagery teaching aids used in this study had a positive impact on learning. Furthermore, an analysis of covariance showed no significant gender differences in learning outcomes between male and female students.

This study demonstrates that children’s learning processes, in addition to relying on individual physiological maturation, can be greatly enhanced through interactions with their environment, thereby fostering intrinsic motivation. This interaction gradually strengthens their understanding of visual images and spatial models, along with their cognitive structures and psychological thinking. The findings align with Bruner’s representational systems theory, particularly the interactive relationship among the three stages of cognitive development. Using teaching aids as structured learning tools enables children to explore and develop intuitive thinking, facilitating the integration of visual–motor skills and reinforcing conceptual connections within cognitive learning. This process effectively helps children establish a solid foundation for further learning.

By engaging with the visual graphic teaching aids, children can integrate movements and perceive differences in the material, size, and shape of objects. They use their fine motor skills such as grasping, holding, rotating, and pulling, which promote visual coordination and control. Teachers observed that children explored structures by manipulating parts through actions such as pulling and stretching, demonstrating improved operational abilities with repeated practice. Additionally, as children observed the images, they learned to appreciate differences in object shapes from various viewing angles, enabling them to identify and control visual–motor integration. They also developed the ability to understand the relative positions of subjects and objects, using themselves as a reference point to recognize and manipulate objects. To sum up, when children play with the teaching aids, they are not only observing the movements produced by objects, they are also utilizing their recognition and understanding of images and movements. During this process, children may pause to think or repeat certain actions to coordinate the relationship between their thoughts and actions.

Teachers also noted that when children operated the visual imagery teaching aids, they initially demonstrated an egocentric perspective regarding the shape, size, distance, and location of objects. However, with repeated practice, they gradually developed an understanding of fundamental concepts related to the relative positioning and spatial arrangement of the main components of the teaching aids. As children enter kindergarten, they begin interacting more with their peers, which is a crucial aspect of social development. Through learning to communicate with others, children acquire an understanding of social roles, expectations, and norms, as well as the importance of cooperation, mutual assistance, and responsibility—key skills necessary for adapting to future social life. Ladd et al. (1999) have also emphasized that social competence in preschoolers is a strong predictor of school adjustment and behavioral outcomes during school age and adolescence (Sahin and Ates 2023).

### 5.3. Learning the Process of Using Visual Image Teaching Aids

This study utilizes visual imagery teaching aids to explore children’s cognitive learning and fine motor skill development. When young children begin engaging with these operations, they often overlook structural clues within visual images, particularly if they lack prior experience with visual image cues. Some children may also struggle to express their ideas independently. In such cases, teachers provide support through verbal and action-based cues, enabling children to organize information, retrieve memories, and build understanding using both images and language. This learning process allows young children to practice handling complex cognitive concepts, thereby enhancing their learning outcomes. From a social constructivist perspective, dialog within social interactions plays a crucial role in promoting the development of a child’s zone of proximal development. Learning scaffolding acts as a bridge that facilitates the connection between cognition and learning, thereby fostering the internalization of skills during social interactions. During teacher–student collaborative activities, “educational dialogue” serves as a scaffold for children’s thinking, guiding their observation and learning processes. In this context, the teacher’s role is not only to help children distinguish between essential and non-essential characteristics, but also to assist in connecting conceptual knowledge. By guiding children to identify problem-solving clues during the thinking process, instructional conversations become more effective in promoting meaningful learning (Ladd et al. 1999; Chou 2017).

In conclusion, the use of visual imagery teaching aids fosters increased opportunities for social interaction and verbal communication among children. During interactions with teachers, peers, and within the learning environment, students are not passive recipients, but active participants who explore and solve problems, thereby co-constructing knowledge.

### 5.4. Recommendations

In this study, we observed that when children engaged with the teaching aids, they tended to observe, imitate, experiment, and interact with their peers. Children with more advanced abilities often took on roles such as asking questions, demonstrating, explaining, guiding, and offering suggestions. This interaction provided a scaffolding effect for children with lower abilities, facilitating problem-solving and self-improvement, which ultimately enhanced their learning outcomes. It is recommended that future research further investigate the oral interactions of young children during problem-solving activities, as well as the reasoning processes involved in information processing and acquisition across different age groups during visual image learning. Such research could offer deeper insights into the strategies and methods children use to approach problem solving. The embedded visual imaging teaching aids in this study demonstrated significant effectiveness in supporting the development of children’s cognitive and fine motor skills. For future studies, researchers could develop more comprehensive assessment methods and conduct follow-up research focusing on teaching methods and instructional strategies.

## Figures and Tables

**Table 1:** Bruner’s theory of cognitive representation systems.

Development Installment	Learning Content
Enactive representation	Toddlers explore through movement to learn about their surroundings and encode and store the perceived movement messages in their memories, laying the foundation for learning.
Iconic representation	Young children learn about the world around them through exploratory experiences and learning that generates perceptual experiences and mental images, and cognitive learning through representational constructions.
Symbolic representation	Young children learn through symbols, words, and language. Language can be used to encode information received and to help construct and think about cognitive concepts.

**Table 2:** Visual action-related assessment tools.

Assessment Tools	Assessment Age	Assessment Ability	Assessment Time	Assessment Method
The Berry-Buktenica Developmental Test of Visual–Motor Integration (VMI)	2–18 years old	Draw 27 geometric figures in order of difficulty with pen and paper	10–15 min	Individual testing
Movement Assessment Battery for Children for Children, Second (Movement ABC)	3–16 years old	Hand operation Ball skills Balance ability	20–30 min	Individual testing
Test of Visual Motor Skills (TVMS)	2–13 years old	Graphic copying	20–30 min	Individual testing
Peabody Developmental Motor Scales (PDMS)	0–6 years old	Reflection Shift Grasp visual–motor coordination	20–30 min	Individual testing

**Table 3:** Design Framework of visual image teaching aids and graphic design strategies in the study.

Gestalt Theory	Design Principles	Teaching Focus
Similarity A combination of image elements with similar characteristics, either as a group or as a whole.	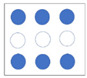	Children are provided with guidance to facilitate their recognition of features through identifying similarities among parts. They are also encouraged to identify the features and similarities between shapes or symbols, promoting their awareness of graphic patterns and norms.
Proximity Graphic elements and closely spaced or related blocks are treated as a group or as a whole.	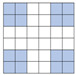	Children are directed to observe and examine the characteristics and positions of images, fostering their ability to recognize similar images as part of the same space or whole through engaging in classification and categorization activities.
Continuity An image is presented with a continuous effect of movement or character, or in a regular pattern that leads the eye to make a connection.	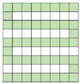	Children are provided with guidance to observe and analyze the representations of continuity within visual sequences of images. This practice enables them to develop their ability to recognize relationships between images, collect relevant information, and further organize the logical structure of the information. These skills serve as a foundation for judgment and critical thinking.
Closure An image that characterizes elements that are closed or an image that causes the eye to perceive the composition of separate elements as a whole.	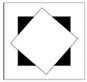	Children are guided to focus on features that belong to each other or that are closed between images to help recognize complex shapes and can practice recognizing changes in the shape of objects for manipulative learning.
Figure-ground The theme of the image is separated from the surrounding area, and the foreground is more prominent, so that different perceptions are created by different focuses of attention.	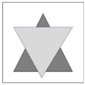	Children are guided to focus their attention on the target to distinguish individual and separate elements of the image through the foreground and background of the image and to integrate the boundaries between the two and their separate forms to facilitate the organization and recognition of the image.
Common Fate Patterns in which patterns and elements of an image are dynamically oriented so that the visual sense perceives them as a whole.	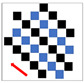	Children are guided to explore the directionality of visual images in relation to their position and to observe the position of the images, and to transform manipulation and movement in the same direction and dynamic.
Symmetry Stability and symmetry in the presentation of an image so that the eye naturally perceives it as a whole.	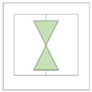	Children are guided to observe the graphic symmetry of visual images so that the operating parts and blocks can show symmetry and stability and can operate according to the sequence and steps of the parts provided in the teaching aids.
Common Region Principle Images are shown in a common region that presents the image’s regional boundaries, but is treated as an individual group.	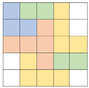	Children are guided to observe images in terms of colors or lines and are able to practice recognizing colors in marked areas, treating common blocks as a group, and using hand–eye coordination to make arrangements and adjustments.

**Table 4:** Summary table of factor analysis.

Title	Content	Factor I	Factor II
10	Teaching toys can help toddlers learn thinking and problem-solving skills.	0.73	
8	Teaching toys can satisfy children’s curiosity to explore and learn.	0.66	
12	Teaching toys are educational, practical, reusable and economical.	0.64	
1	Teaching toys designed with visual graphics and symbols with creative designs.		0.87
11	Teaching toys can provide visual image cognitive learning.		0.86
5	Teaching toys are designed with diversified and rich ways of play to enhance children’s learning enjoyment.		0.76
6	Teaching toys can provide different visual and tactile stimulation and learning for toddlers.		0.69
4	Teaching toys can enhance the fine motor development of toddlers.		0.63
7	Teaching toys can develop children’s self-care skills.		0.57
Earmark		6.44	1.23
Variation (%)		53.68	10.21
Cumulative Interpretable Variables (%)		53.68	63.89

**Table 5:** Descriptive statistics of characteristics of teaching aids.

Question	Group	Traditional Teaching Aids		Visual Graphic Aids	
	Content	Mean	Standard Deviation	Mean	Standard Deviation
9	Connecting teaching toys with children’s life experiences to arouse children’s interest in learning.	1.90	1.90	4.86	0.35
2	The overall design of teaching toys ranges from easy to difficult, and the gradual progression can guide children to explore learning opportunities.	2.53	0.81	4.90	0.35
3	Teaching toys provide hands-on learning opportunities for young children.	2.69	0.84	4.91	0.28
10	Teaching toys can help toddlers learn thinking and problem solving skills.	1.76	1.07	4.87	0.34
8	Teaching toys can satisfy children’s curiosity to explore and learn.	1.90	1.08	4.86	0.35
12	Teaching toys are educational, practical, reusable and economical.	1.77	1.07	4.91	0.28

**Table 6:** Descriptive statistics of functions of teaching aids.

Question	Group	Traditional Aids		Visual Graphic Aids	
	Content	Mean	SD	Mean	SD
1	Creative Designs for Teaching Toys.	2.76	0.71	4.94	0.23
4	Teaching toys can enhance the development of fine motor skills of young children.	2.24	0.94	4.91	0.28
5	The design of educational toys is diversified, which enhances the enjoyment of learning in young children.	1.91	0.96	4.89	0.32
6	Teaching toys can provide toddlers with different visual and tactile stimulation.	2.01	0.91	4.84	0.37
7	Teaching toys can cultivate children’s self-care ability.	1.96	1.08	4.87	0.34
11	Teaching toys can provide visual image cognitive learning.	1.86	1.11	4.93	0.31

**Table 7:** Results of one-way ANOVA for characteristics of educational toys by background variables.

Background Variables	Age	Count	Mean	SD	F
Age	(1) 21–30	21	4.90	0.18	0.10
	(2) 31–40	22	4.88	0.19	
	(3) 41–50	27	4.88	0.29	
Education level	(1) No university degree	23	4.88	0.19	0.05
	(2) University	47	4.89	0.25	
Type of facility	(1) Kindergarten	63	4.88	0.24	1.21
	(2) Infant care centers	7	4.98	0.63	
Years of experience	(1) Less than 5	35	4.93	0.15	2.49
	(2) 5 years and above	35	4.84	0.28	
Age of children	(1) 2–3	9	4.96	0.11	1.32
	(2) 3–4	24	4.90	0.27	
	(3) 4–5	23	4.91	0.17	
	(4) 5–6	14	4.89	0.28	

**Table 8:** Results of one-way ANOVA for functions of educational toys by background variables.

Background Variables	Age	Count	Mean	SD	*F*
Age	(1) 21–30	21	4.94	0.13	0.80
	(2) 31–40	22	4.91	0.18	
	(3) 41–50	26	4.86	0.29	
Education level	(1) No university degree	23	4.94	0.11	1.42
	(2) University and above	47	4.88	0.25	
Type of facility	(1) Kindergarten	63	4.89	0.22	0.15
	(2) Infant care centers	7	4.93	0.19	
Years of experience	(1) Less than 5	35	4.95	0.12	4.63 *
	(2) 5 years and above	35	4.84	0.28	
Age of children	(1) 2–3	9	4.98	0.56	1.26
	(2) 3–4	24	4.90	0.25	
	(3) 4–5	23	4.91	0.14	
	(4) 5–6	14	4.81	0.31	

* *p* < 0.05.

**Table 9:** Mean scores and standard deviations of the pre-test and post-tests of the learning assessment of visual aids in this study.

Measurement	Pre-Test		Post-Test		t	Cohen’s d
	Mean	SD	Mean	SD		
Patten recognition	3.10	0.28	4.48	0.47	22.70 *	0.43
Patten understanding	2.89	0.27	4.26	0.34	24.04 *	0.40
Spatial thinking	3.00	0.17	4.43	0.47	26.78 *	0.49
Fine motor moments	3.11	0.27	4.62	0.44	24.92 *	0.49

* *p* < .05.

**Table 10:** Homogeneity of regression coefficients within the visual image learning scale in this study.

Measurement	F-Value	*p*-Value
Patten recognition	1.13	.289
Patten understanding	0.43	.514
Spatial thinking	0.21	.651
Fine motor moments	0.36	.549

**Table 11:** Covariate analysis of visual image learning scales in this study.

Measurement Items	Source of Variation	*SS*	*df*	*MS*	F-Value	*p*-Value
Patten recognition	pre-test	2.261	1	2.261	12.163 **	.001
	Gender		1		0.000	.988
Patten understanding	pre-test	0.175	1	0.175	1.501	.226
	Gender	0.025	1	0.025	0.217	.643
Spatial thinking	pre-test	0.093	1	0.093	0.409	.525
	Gender	0.237	1	0.237	1.045	.311
Fine motor moments	pre-test	0.140	1	0.140	0.713	.402
	Gender	0.035	1	0.035	0.176	.676

** *p* < 0.01.

## Data Availability

The data presented in this study are available on request from the corresponding author. The data are not publicly available due to privacy.
